# Chronic Unilateral Vestibular Hypofunction: Insights into Etiologies, Clinical Subtypes, Diagnostics and Quality of Life

**DOI:** 10.3390/jcm13185381

**Published:** 2024-09-11

**Authors:** Mustafa Karabulut, Wolfgang Viechtbauer, Lien Van Laer, Alfarghal Mohamad, Vincent Van Rompaey, Nils Guinand, Angélica Perez Fornos, Marie-Cecile Gerards, Raymond van de Berg

**Affiliations:** 1 Division of Vestibular Disorders, Department of Otorhinolaryngology and Head and Neck Surgery, School for Mental Health and Neuroscience, Maastricht University Medical Center, 6229HX Maastricht, The Netherlands; 2 Department of Psychiatry and Neuropsychology, Maastricht University, 6229HX Maastricht, The Netherlands; 3 Department of Rehabilitation Sciences and Physiotherapy/Movant, Faculty of Medicine and Health Science, University of Antwerp, 2000 Antwerp, Belgium; 4 Multidisciplinary Motor Centre Antwerp (M2OCEAN), University of Antwerp, 2000 Antwerp, Belgium; 5 Department of Ear Nose Throat, King Abdul Aziz Medical City, Jeddah 22384, Saudi Arabia; 6 Department of Otorhinolaryngology and Head & Neck Surgery, Antwerp University Hospital, Faculty of Medicine and Health Sciences, University of Antwerp, 2000 Antwerp, Belgium; 7 Service of Otorhinolaryngology Head and Neck Surgery, Department of Clinical Neurosciences, Geneva University Hospitals, 1205 Geneva, Switzerland

**Keywords:** unilateral vestibular hypofunction, etiology, secondary diagnoses, clinical subtypes, quality of life

## Abstract

**Background/Objectives**: Chronic unilateral vestibular hypofunction (UVH) can lead to disabling vestibular symptoms and a decrease in quality of life. The aim of this study was to investigate etiologies, clinical subtypes, symptoms, and quality of life (QoL) in patients with chronic UVH. **Methods**: A retrospective study was performed on 251 UVH patients in a tertiary referral center. Inclusion criteria comprised reduced or absent caloric responses, with a caloric asymmetry ratio ≥25%. Patients with central vestibular pathology, symptom duration <3 months, and incomplete responses to questionnaires were excluded. Patient records were assessed for etiologies, secondary vestibular diagnoses, clinical subtypes, and questionnaires related to QoL. Additionally, multiple linear regression analysis was performed to evaluate factors influencing QoL. **Results**: Thirteen different etiologies were identified, with Menière’s Disease as the most prevalent (31%, *n* = 79). The most frequently reported secondary vestibular diagnoses were benign paroxysmal positional vertigo (BPPV) (21%, *n* = 54) and persistent postural perceptual dizziness (PPPD) (19%, *n* = 47). Five distinct clinical subtypes were identified: recurrent vertigo with UVH (47%), rapidly progressive UVH (25%), idiopathic/unknown UVH (18%), slowly progressive UVH (8%), and congenital UVH (2%). Over 80% of UVH patients experienced moderate-to-severe handicap, as indicated by the Dizziness Handicap Inventory. Approximately 20–25% of UVH patients exhibited moderate-to-severe depression and anxiety, based on the Hospital Anxiety and Depression Scale. Multiple linear regression analyses demonstrated that the presence of PPPD significantly reduced QoL in chronic UVH patients. **Conclusions**: Chronic UVH is a heterogeneous disorder. Secondary vestibular diagnoses like BPPV and PPPD often co-exist and can significantly impact QoL. A structured diagnostic approach and tailored interventions are crucial to address the diverse needs of UVH patients.

## 1. Introduction

Unilateral vestibular hypofunction (UVH) is a complex disorder characterized by a unilaterally decreased or absent vestibular function, affecting the vestibular end-organ, vestibular nerve, or both. Patients may experience a range of symptoms [1,2,3,4]. When UVH occurs, the central nervous system attempts to decrease symptoms via a process called vestibular compensation [5,6]. However, its effectiveness can be influenced by various factors such as age, the etiology of vestibular hypofunction, the presence of co-morbidities, psychological factors such as depression or anxiety, environmental factors, level of physical activity, and individual variability to adapt and compensate [7,8,9,10]. UVH can therefore result in chronic symptoms like chronic dizziness and imbalance [3,11]. Despite being widespread, the exact prevalence of UVH remains uncertain due to its diverse presentation. Thus, the impact of UVH remains controversial and is often underestimated. Nevertheless, chronic UVH (present for > 3 months) reduces quality of life (QoL) and imposes a significant socioeconomic impact [3,12].

UVH can have different etiologies such as Menière’s Disease (MD), infection/inflammation, Vestibular Migraine (VM), vascular, iatrogenic, neoplasm, and trauma [3,13]. Identifying the etiology can be challenging due to its heterogeneous nature, especially since the symptoms might overlap with other vestibular disorders. This complexity requires thorough evaluation. Moreover, UVH can present with different clinical subtypes, including recurrent vertigo with UVH, rapidly progressive UVH, slowly progressive UVH, and idiopathic/unknown UVH [14,15]. These variations complicate the identification of its exact cause, thereby hindering accurate diagnosis.

Diagnosing UVH can be challenging, especially since no standardized diagnostic criteria are currently available. Therefore, UVH is frequently under- or misdiagnosed [16,17]. Various clinical diagnostic tests can be used for vestibular assessment: the caloric test, rotatory chair test, (video) head impulse test (vHIT), vestibular evoked myogenic potentials, dynamic visual acuity, etc. [18,19]. Additional tests, such as audiological assessment and imaging, may be used to help with the diagnosis and identify potential underlying causes [20]. Although many of these tests provide objective information about the vestibular and audiological function, they may not fully capture the complete picture of patients’ overall functional health status [21,22,23,24].

Chronic UVH can decrease QoL. One in three UVH patients (with chronic symptoms) still continues to experience significant handicap [3]. This may not only result from inadequate vestibular compensation, but also from environmental factors, psychological conditions, and functional problems such as Persistent Postural-Perceptual Dizziness (PPPD) [25,26,27,28,29]. Therefore, evaluating QoL is valuable, since it enables a more comprehensive evaluation of the impact of chronic UVH [30,31,32]. However, it is important to mention that questionnaires currently used to evaluate the impact of vestibular deficits on QoL, like the Dizziness Handicap Inventory (DHI), were not specifically developed for UVH. This might impede the thorough evaluation of UVH patients before and after therapeutic interventions. In this context, a patient-reported outcome measure (PROM) is needed for patients with chronic UVH.

As such, further research is required to explore potential etiologies of chronic UVH, and to fully understand the clinical manifestations and quality of life in UVH patients. This retrospective study therefore aimed to investigate the etiologies, clinical subtypes, symptoms and QoL in patients with chronic UVH. The findings from this study could potentially contribute to the development of diagnostic criteria for chronic UVH.

## 2. Materials and Methods

### 2.1. Ethical Approval

The study protocol was approved as non-WMO (Wet Medisch-Wetenschappelijk Onderzoek) research by the medical ethical testing committee of the University Maastricht/academic hospital Maastricht (UM/MUMC+) as well as receiving consent from the advisory board of the MUMC+ (METC 2021-2936 and date of approval 28 December 2022). This study followed the guidelines outlined by Dutch legislation.

### 2.2. Participants

**Figure 1:**
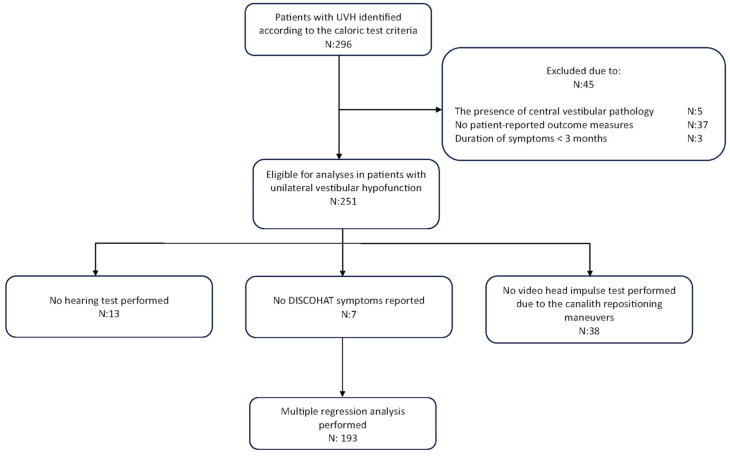
A flowchart of our retrospective study design.

This retrospective study involved patients diagnosed with UVH by two of the authors (MCG, RvdB) at a tertiary referral center, between 2017 and 2021. UVH patients were included based on reduced or absent responses during bithermal caloric irrigation, characterized by a caloric asymmetry ratio ≥25% between ears [33], where the absolute value of the healthy side was within the range of 25°/s to 83°/s (sum of the mean slow-phase eye velocity in both cold and warm irrigations). Patients with central vestibular pathology, incomplete responses to questionnaires related to quality of life, and symptom duration < 3 months were excluded from the study (Figure 1).

### 2.3. Etiology and Clinical Subtypes

The medical history of the patients was analyzed from their files to identify the etiology of UVH and possible secondary vestibular diagnoses (conducted by MCG). The diagnoses of MD [34], VM [35], benign paroxysmal positional vertigo (BPPV) [36], and PPPD [37] were established according to the Bárány Society’s Classification Committee criteria. Each etiology was assigned one of three levels of certainty: definite, probable, or idiopathic/unknown.

Etiologies of UVH were classified into different clinical subtypes:

- Recurrent vertigo with UVH—This subtype involved recurrent, brief vertigo attacks. Patients may have experienced vertigo, imbalance and/or oscillopsia (an illusion of an unstable vision). Conditions linked to this subtype included MD, VM, BPPV, benign recurrent vertigo, auto-immune inner ear disease, and overlap MD/VM;
- Rapidly progressive UVH—Patients in this subtype experienced a sudden onset of UVH symptoms, which rapidly progressed. Conditions could include infection/inflammation (like acute unilateral vestibulopathy/vestibular neuritis, labyrinthitis), trauma (labyrinthine concussion), vascular (inner ear ischemia), and iatrogenic factors such as stapedotomy or intratympanic/systemic gentamicin administration;
- Slowly progressive UVH—Clinical symptoms of UVH developed gradually in this subtype, typically without episodes of vertigo. Conditions associated with this subtype included vestibular schwannoma, cholesteatoma, retrofenestral otosclerosis, labyrinthitis ossificans, and iatrogenic factors such as radiation therapy;
- Idiopathic/unknown UVH—In this subtype, the dizziness complaints or vertigo attacks could not be attributed to any specific vestibular disorder or trauma. However, patients exhibited symptoms of vestibular hypofunction (e.g., imbalance, dizziness with fast head movements) despite never experiencing any specific episodes.
- Congenital UVH—This subtype refers to UVH caused by genetic factors, such as inner ear malformations.

Patients were further categorized based on the presence of attacks. Four different categories were established: no attack, one attack, previous attacks, and current attacks. Clinical subtypes without attacks, namely, the slowly progressive UVH and idiopathic/unknown UVH subtypes, were combined and categorized as “no attack”. Patients categorized with rapidly progressive UVH experienced “one attack”. Patients experiencing recurrent vertigo with UVH were divided into “current attacks” and “previous attacks” based on a two-month duration cut-off value.

### 2.4. Symptoms and Co-Morbidities

At the tertiary referral center of this study, templates for medical history taking are based on the 4-step approach [38]. This systematic method facilitates the structured evaluation of various symptoms and co-morbidities. DISCOHAT symptoms, as previously described [11], were identified through history-taking. Briefly, “DISCOHAT” is an acronym used to categorize chronic vestibular symptoms. This includes darkness worsens symptoms, imbalance, supermarket effect, cognitive complaints, oscillopsia, head movements worsen symptoms, autonomic complaints and tiredness. Patients were further categorized into three groups based on symptom duration: 3–12 months, 12–24 months, and ≥24 months. This categorization made it possible to investigate how different durations of symptoms were related to QoL scores. Co-morbidities such as migraine headaches, non-migraine headaches, hypertension, diabetes, depression, anxiety, autoimmune disorders, substance use, and cognitive decline were investigated within the UVH patient cohort. The presence of symptoms and co-morbidities were classified as “present” and “missing data” based on available patient information.

### 2.5. Objective Vestibular Testing

All patients underwent vestibular testing including bithermal caloric irrigation and vHIT. These tests were conducted by trained laboratory technicians following standardized protocols [20,39]. The caloric test was performed with warm (44 °C) and cold (30 °C) water irrigations, 300 mL each, in a dark room. The caloric asymmetry was determined using the Jongkees’ formula. The vHIT was conducted using the ICS Impulse (GN Otometrics; Taastrup, Denmark) and EyeSeeCam (Interacoustics VOG; Munich, Germany). This test involved unpredictable, rapid (>150°), and low-amplitude (±20°) head movements in the horizontal plane, while the patient visually fixated on an earth-fixed target. At least seven head impulses had to be correctly performed in both directions. The horizontal vestibulo-ocular reflex gain for each direction was calculated by the software.

### 2.6. Ancillary Testing

Non-vestibular diagnostic procedures included pure tone audiometry, computed tomography (CT), and magnetic resonance imaging (MRI). Pure tone audiometry included the collection of modified Fletcher indexes (FI) and the type of hearing loss. The degree of hearing loss based on modified FI was determined using the criteria established by the American Speech–Language–Hearing Association (ASHA) [40]. Findings from both MRI and CT scans have been documented.

### 2.7. Quality of Life and Symptoms (QoL)

The QoL was assessed using digital questionnaires: Dizziness Handicap Inventory (DHI), Hospital Anxiety and Depression Scale (HADS), and the European Quality of Life 5 Dimension 5 Level (EQ-5D-5L). The DHI is a validated self-report questionnaire consisting of 25 questions that quantify the impact of dizziness on daily life. Patients respond to each question with “yes”, “sometimes”, or “no”, and each response is assigned a score of four, two, or zero points, respectively. The total DHI score reflects the self-perceived level of dizziness handicap, categorized as mild (0–30), moderate (31–60), or severe (61–100) [41,42]. The HADS assesses the presence of anxiety and depression levels in patients. Scores ranging from 0 to 7 indicate “normal”, 8 to 10 “mild”, 11 to 14 “moderate”, and 15 to 21 “severe” levels of anxiety or depression [43]. The EQ-5D-5L consists of two sections: the EQ-5D descriptive system and the EQ Visual Analog Scale (VAS). While the former evaluates the patient’s health state across five dimensions—mobility, self-care, usual activities, pain/discomfort, and anxiety/depression—the latter, the EQ VAS, is used to assess the patient’s self-rated overall health condition [44,45]. Regarding the EQ-5D-5L, only the VAS was used in the analysis of this study.

### 2.8. Statistical Analysis

Data were analyzed using both visual (histogram and Q-Q plots) and statistical (Shapiro–Wilk test) methods. Results are expressed as mean ± standard deviation for normally distributed data and as median (Interquartile range = IQR) for non-normally distributed data. Bar plots and scatter plots have been used to depict frequency distributions and the relationship between variables, respectively. To quantify the association between variables, Pearson correlations and corresponding tests were used. Multiple linear regression analyses were conducted to investigate the predictors influencing distinct dimensions of QoL in patients with UVH. Predictors included presence of PPPD, migraines, hearing status, duration of symptoms, the presence of attacks, and vestibular test results. The presence of PPPD and migraines were coded as binary variables (yes/no). Hearing status was treated as a continuous variable and coded such that an increase in the hearing score corresponded to an increase in the degree of hearing loss. The duration of symptoms and attacks were categorical variables, as previously described. A subgroup analysis categorized caloric test results into three groups: ≥25°/s, ≥6°/s and <25°/s, and <6°/s. vHIT was not included in the subgroup analysis due to its potential to introduce selection bias (e.g., vHIT was not performed in BPPV patients). Any patients with missing data related to these factors were excluded from the analysis, which resulted in a final cohort of 193 patients (Figure 1). For factors with three or more levels, omnibus F-tests were used to first assess the presence of any differences between groups, before interpreting individual group contrasts. A significance level of 0.05 was used for the statistical analyses. All analyses were conducted using R (version 4.2.2).

## 3. Results

A total of 251 patients were included in the study. The mean age of patients at the time of diagnosis was 59 ± 13 years, with age ranging from 18 to 84 years. Among the study population, 58% were female and 42% were male. The patient characteristics are presented in Table 1.

### 3.1. Etiology

**Figure 2:**
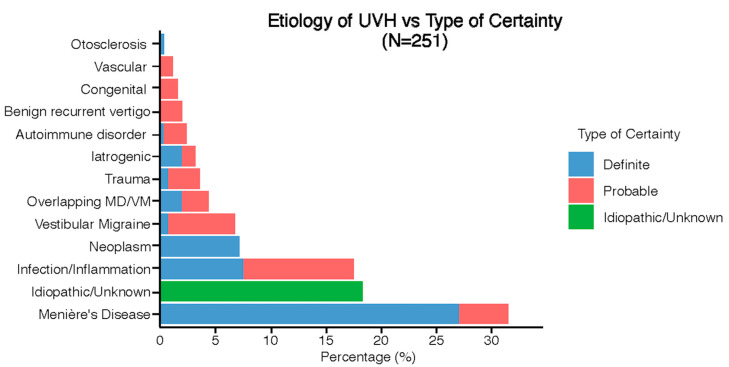
Distribution of etiologies in the UVH population divided into type of certainty.

Figure 2 presents the etiologies of UVH patients, classified by diagnostic certainty. Thirteen different etiologies were identified, with Menière’s Disease as the most prevalent (31%, *n* = 79). A definite etiology of UVH was established in 48% of cases (n = 121), while a probable etiology was identified in 34% (*n* = 84). The etiology of UVH remained unknown/idiopathic in 18% of cases (*n* = 46). More details about etiologies of UVH can be found in Table S1.

**Figure 3:**
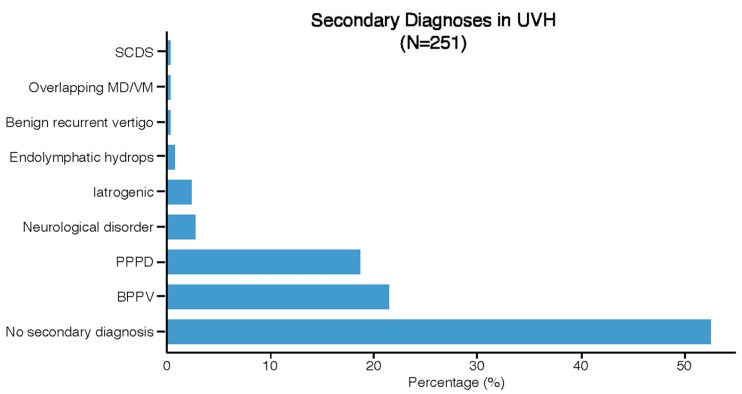
Distribution of secondary diagnoses in the UVH population.

Figure 3 presents the secondary diagnoses in UVH patients. Secondary diagnoses were identified in 119 UVH patients. The most frequently reported secondary diagnoses were BPPV (21%, *n* = 54) and PPPD (19%, *n* = 47). BPPV was identified as a secondary diagnosis in patients with the following etiologies: idiopathic/unknown UVH (*n* = 23), infection/inflammation (*n* = 10), MD (*n* = 6), trauma (*n* = 5), and iatrogenic (n = 4). PPPD was given as a secondary diagnosis in patients with the following etiologies: infection/inflammation (*n* = 20), MD (*n* = 11), VM (*n* = 5), overlap MD/VM (*n* = 5), and idiopathic/unknown (*n* = 4).

### 3.2. Clinical Subtypes of UVH

**Figure 4:**
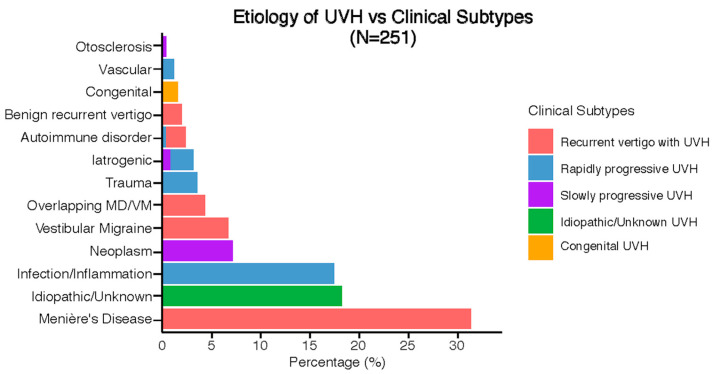
Clinical subtypes of UVH with respect to the etiologies.

Figure 4 illustrates the previously discussed etiologies subdivided into clinical subtypes. Five distinct clinical subtypes were identified: recurrent vertigo with UVH (47%, *n* = 117), rapidly progressive UVH (25%, *n* = 63), idiopathic/unknown UVH (18%, *n* = 46), slowly progressive UVH (8%, *n* = 21), and congenital UVH (2%, *n* = 4). Most etiologies only had one clinical subtype (e.g., Menière’s Disease was always paired with recurrent vertigo with UVH), while only iatrogenic and autoimmune disorders presented with different clinical subtypes.

### 3.3. Co-Morbidities and Vestibular Symptoms

Eight different co-morbidities were investigated. The most prevalent were non-migraine headaches (36%, *n* = 91) and hypertension (34%, *n* = 86) (Figure S1). Regarding DISCOHAT symptoms, imbalance was most common (70%, *n* = 175), whereas oscillopsia was the least reported (22%, *n* = 57) (Figure S2).

### 3.4. Imaging

Cerebral imaging was documented in 82% of patients (*n* = 168 MRI; *n* = 37 CT). Abnormalities were detected in 17% (*n* = 41) through MRI scans and in 4% (*n* = 11) through CT scans. Vestibular schwannoma was the most frequently reported MRI abnormality, whereas superior semicircular canal dehiscence (SCD) and trauma/skull base fracture were most frequently demonstrated on CT scans. In total, 61% of patients (*n* = 153) exhibited no abnormalities, while 18% (*n* = 46) did not require imaging (Table 2).

### 3.5. Hearing

Pure tone audiometry was conducted on 238 patients. Among them, 64% (*n* = 152) showed either normal hearing or slight to moderate hearing loss in both ears, with a modified Fletcher Index ranging from -10 to 55 decibels. No hearing loss was detected in 17% of the patients (*n* = 40). Additionally, 52% of the patients demonstrated asymmetric hearing loss. The detailed results of the pure tone audiometry are summarized in Table S2.

### 3.6. Vestibular Testing

Based on the caloric test, 138 patients had UVH on the right side, while 113 patients had UVH on the left side. The caloric asymmetry analysis revealed a mean score of 47.71 ± 19.99 for the right side and 53.39 ± 21.45 for the left side. When examining the vHIT gain, the side affected by the caloric test had a median gain of 0.88 (0.20), slightly lower than the non-affected side, which had a median gain of 0.89 (0.14) (*p* > 0.05). The correlation between the caloric asymmetry and the vHIT asymmetry was moderately significant (r = 0.44; *p* < 0.001) (Figure S3).

### 3.7. Quality of Life (QoL) and Psychological Symptoms

More than 80% of UVH patients experienced moderate-to-severe handicap, as indicated by the DHI. The DHI showed a mean score of 51.63 ± 21.61, indicating a moderate handicap. The functional subscale had the highest score (20.39 out of max. 36 points). Approximately 20–25% of UVH patients reported moderate-to-severe depression and anxiety, based on the HADS. The HADS showed median scores regarding anxiety and depression (depression = 6 (IQR 7.5), anxiety = 6 (IQR 6.5)). The EQ-5D-5L demonstrated a median VAS score of 65 (IQR 30) (Table 3).

### 3.8. Vestibular Testing and QoL

Weak positive correlations were found between caloric asymmetry and DHI total scores (r = 0.14, *p* = 0.02), as well as between caloric asymmetry and HADS depression scores (r = 0.12, *p* = 0.04). No significant correlation was found between caloric asymmetry and HADS anxiety scores, or between caloric asymmetry and EQ-5D-5L scores (both *p* > 0.05) (Figure S4). Similarly, no significant correlation was observed between vHIT asymmetry and questionnaires related to QoL (all *p* > 0.05) (Figure S5). Additional correlation analyses were conducted within questionnaires. Strong positive correlations were observed between physical and functional subscales of the DHI (r = 0.74; *p* < 0.001), as well as between the functional and emotional subscales (r = 0.72; *p* < 0.001). Moderate positive correlations were found between DHI functional subscales and both HADS depression and anxiety scores (r = 0.55 and 0.49; *p* < *0*.001, respectively). Weak positive correlations were found between the DHI physical score and both HADS depression and anxiety scores (r = 0.39 and 0.38; *p* < *0*.001, respectively) (Figure S6).

### 3.9. Factors Influencing Quality of Life and Symptoms

Seven different models were fitted using multiple linear regression analyses to investigate factors related to the various QoL outcomes (questionnaires related to QoL). Across the models (Table 4), the PPPD variable exhibited a significant relationship with the QoL outcomes, indicating that the presence of PPPD had an adverse impact on QoL. Patients with previous vertigo attacks reported significantly lower DHI functional and HADS anxiety scores, compared to those with no attacks. Caloric test results <6°/s exhibited significant negative associations with DHI physical and functional subscores, and total DHI scores (*p* < 0.05), when compared to caloric test results ≥25°/s. Migraine and duration of symptoms were not significantly related to QoL. Hearing loss was positively associated with DHI functional subscores (*p* = 0.04), indicating that higher levels of hearing loss resulted in poorer functional quality of life. Overall, the models showed varying levels of predictive power (adjusted R-squared values ranging from 0.04 to 0.21).

## 4. Discussion

In this study, 251 patients with chronic UVH were evaluated regarding etiologies, secondary diagnoses, clinical subtypes, vestibular and psychological symptoms, and quality of life. Thirteen different etiologies were identified, with MD being the most prevalent. The most frequently reported secondary vestibular diagnoses were BPPV and PPPD. Five different clinical subtypes were identified, varying from recurrent vertigo attacks to idiopathic without attacks. Imbalance was the most predominant symptom, while oscillopsia was the least frequently reported symptom in UVH patients. The impact on quality of life was profound, and over 80% experienced moderate to severe handicap, as measured by the DHI. Moreover, approximately 20–25% of UVH patients demonstrated moderate to severe levels of depression and anxiety, based on the HADS. The presence of PPPD was associated with a significant reduction in QoL among chronic UVH patients.

### 4.1. Etiologies

MD was identified as the most common etiology of UVH (31%), consistent with a previous systematic review [3]. However, the prevalence of UVH etiologies varies in the literature. This might be related to several factors. One such factor is the variability in diagnostic criteria for UVH. For example, in a study of 302 patients, acute unilateral peripheral vestibulopathy and vestibular schwannoma were identified as the primary etiologies when UVH was diagnosed using vHIT criteria [46]. On the other hand, when UVH was diagnosed based on caloric testing criteria, MD cases were more frequently identified [47,48]. This dissociation between the vHIT and caloric test could (partially) be explained by the presence of hydrops [49]. Selection bias is another important consideration. For instance, ENT surgeons often see patients with hearing loss. This could increase the frequency of vestibular disorders in which hearing loss and vestibular symptoms are combined (e.g., Menière’s Disease, genetic inner ear disorders, etc.). Furthermore, tertiary referral centers encounter a different patient population than primary care providers. To summarize, the prevalence of etiologies in UVH patients depends on the diagnostic criteria and the clinical setting.

### 4.2. Secondary Diagnoses

The most frequent secondary diagnoses in this UVH population were BPPV (21%) and PPPD (19%). BPPV, in particular, is known to co-exist with various pathological conditions [50]. While the lifetime prevalence of BPPV is reported as 2.4% [51], its prevalence as a secondary condition to UVH, such as vestibular neuritis [52,53,54,55] and Menière’s Disease [56,57,58], varies from 1.8% to 22.2% [55]. This wide range indicates the need to consider underlying vestibular conditions when diagnosing UVH. While most patients with vestibular symptoms are diagnosed with a single vestibular disorder, the occurrence of multiple diagnoses ranges from 3.7% [59] to 30.1% [60,61]. Thus, if symptoms persist following BPPV treatment, assessment for other vestibular diagnoses like UVH becomes necessary. After all, BPPV may serve as a potential indicator of vestibular hypofunction [55]. Therefore, the presence of persistent symptoms highlights the need for positional testing, as UVH and BPPV can present with similar symptoms [62], such as dizziness with quick head movements [16].

The prevalence of PPPD was found to range from 19% to 21.8% among three studies conducted within a tertiary vertigo clinic population [63,64,65]. This is consistent with the findings of this study (19%). PPPD can be triggered by both acute and episodic vestibular disorders [37,66,67]. A previous study indicated that patients with a history of multiple vestibular disorders (Menière’s Disease, Vestibular Migraine, BPPV) are at an increased risk of developing PPPD [67]. Therefore, when encountering patients with vestibular hypofunction and a decreased QoL, clinicians should explicitly screen for PPPD.

Preferably, a structured approach is used, such as the four-step approach to history-taking [38]. Screening for PPPD enables healthcare professionals to identify PPPD early on, and to tailor interventions. These interventions may include physical therapy, the treatment of co-existing vestibular and functional disorders, and/or addressing psychological aspects [68]. To summarize, a comprehensive approach that addresses both UVH and PPPD is essential for optimizing patient care and enhancing their QoL.

### 4.3. Clinical Subtypes

The majority of UVH patients exhibited clinical subtypes consistent with their etiology (e.g., Menière’s Disease—recurrent vertigo with UVH), while only iatrogenic and autoimmune disorders presented with different clinical subtypes. Among the idiopathic/unknown UVH patients, no attacks were reported, indicating an overlap in clinical presentations between slowly progressive UVH and idiopathic/unknown UVH. This overlap contributes to the complexity of the diagnostic process. Additionally, these findings show that vertigo does not need to be present as a symptom in patients with UVH.

### 4.4. Co-Morbidities

Non-migraine headache (36%) and hypertension (34%) were the most commonly reported co-morbidities in this UVH population. This is congruent with previous the literature, which showed significant associations of non-migraine headache and hypertension with vestibular disorders [60,69,70,71]. However, the relationship between co-morbidities and UVH is complex. While some can directly contribute to vestibular hypofunction, others arise from underlying pathologies. For example, conditions like hypertension and diabetes can affect vascular health, which in turn may impact the blood supply of the vestibular system. Similarly, depression and anxiety can exacerbate vestibular symptoms through psychological mechanisms. However, these co-morbidities may also co-occur with UVH due to shared risk factors and common underlying pathologies (e.g., depression and anxiety can occur after UVH). Furthermore, autoimmune disorders are commonly associated with UVH. Among the six patients with autoimmune disorders in this study, a causal relationship between UVH and autoimmunity was very likely. Additionally, nine patients with known autoimmune disorders experienced Menière’s Disease and Vestibular Migraine, suggesting a potential association between UVH and autoimmunity. Two patients had idiopathic UVH, potentially linked to autoimmunity. The prevalence of autoimmune disorders in western countries is typically around 8% [72], consistent with the 7% observed in our population. Thus, while autoimmune disorders may not directly cause UVH, they might play a modulating role in its development [73].

### 4.5. Symptoms

Imbalance was the most frequently reported symptom. Therefore, it might be considered the main symptom of UVH. Additionally, the spectrum of other symptoms related to UVH varied from supermarket effect and oscillopsia to cognitive complaints and tiredness. Oscillopsia was reported in 22% of the patients, which is consistent with previous findings [3,11]. This suggests that input from the remaining vestibular organ and central compensation mechanisms (e.g., compensatory saccades) may be sufficient to enable gaze stabilization and maintain dynamic visual acuity. However, it can still fail in a subset of UVH patients.

### 4.6. Imaging

In 61% percent of UVH patients who underwent imaging, no abnormalities were found. Imaging is considered necessary in some diagnostic work-ups (e.g., MRI in Menière’s Disease to rule out a vestibular schwannoma) [34]. Unfortunately, in routine clinical practice, there is still an overuse of imaging in vestibular patients (e.g., BPPV). This increases costs and radiation exposure [74,75,76]. Relying more on history-taking and clinical examination to assess vestibular patients can reduce this overuse and improve healthcare efficiency [38]. Therefore, incorporating clinical guidelines to determine when imaging is necessary for UVH patients could help reduce unnecessary scans.

### 4.7. Vestibular Testing

#### 4.7.1. Caloric Test

Inclusion criteria involved a unilateral caloric asymmetry ratio of ≥25%, along with normal caloric test results on the “healthy” side. This implies that the “healthy” side needed to demonstrate absolute values within the range of 25°/s to 83°/s (sum of bithermal caloric irrigations). While vestibular asymmetry is often regarded as a criterion [11,77,78], the significance of the absolute values tends to be overlooked. For example, patients with a bilateral vestibulopathy might be misdiagnosed as UVH, in cases when the absolute values are not evaluated [79]. Currently, no world-wide consensus is (yet) reached on caloric test criteria for UVH. For caloric test criteria, it would be preferred to take both vestibular asymmetry and absolute values into account.

#### 4.7.2. (Video) Head Impulse Test

vHIT is less sensitive than the caloric test for detecting UVH [80]. This might be (partially) explained by vHIT primarily assessing high-frequency vestibular function, while the caloric test reflects the function of the lower frequencies [80]. Additionally, the presence of endolymphatic hydrops might contribute to the discrepancy between vHIT and caloric test results (see above [49]). Only a moderate positive correlation between both tests was found in this study, congruent with the literature [80,81]. It was previously demonstrated that in most cases, the caloric asymmetry needs to exceed 40–60% in order to become noticeable in vHIT [82,83,84]. Nevertheless, vHIT is a much quicker test with a lower burden than the caloric test. It was therefore proposed to first perform a vHIT, and the caloric test only on indication. In case vHIT is abnormal, no caloric test needs to be performed. However, many vestibular patients have a normal vestibular function. Therefore, 13 vHITs need to be performed, in order to save one caloric test [85].

Finally, vestibular function tests often focus on only one part of the vestibular system (e.g., horizontal canal). The otolith function is frequently not included. Nevertheless, otolith function might be important as otolith dysfunction seems quite prevalent in PPPD [86,87].

### 4.8. QoL and Psychological Symptoms

In a previous review, around 30% of UVH patients were found to have at least a moderate handicap [3]. In this study, more than 80% reported moderate to severe handicap according to DHI scores. Multiple factors could contribute to this difference, such as selection bias (e.g., this study involved a tertiary referral center) and different study designs (systematic review versus retrospective study). Despite this difference, both studies show that a significant amount of UVH patients experience at least a moderate handicap. Furthermore, a subset of UVH patients reported moderate anxiety, consistent with the literature [88,89]. It is therefore imperative to take QoL and psychological/psychiatric factors into account when evaluating UVH patients in clinic [32].

### 4.9. Vestibular Testing and QoL

A weak correlation was found between caloric asymmetry and DHI total scores, which is inconsistent with previous literature [24,84,90,91]. Additionally, as stated above, caloric test results <6°/s exhibited significant negative associations with DHI physical, functional subscores, and total DHI scores. This could imply that severe vestibular hypofunction can decrease patient’s perceived dizziness-related disability. Currently, several vestibular-specific PROM exist, such as the DHI and the vestibular disorders activities of daily living scale (VADL), to evaluate QoL. The DHI evaluates different aspects of vestibular complaints (function, physical, and emotional) and the VADL assesses the independency in activities of daily living. However, both questionnaires are not specifically tailored to UVH. Therefore, there is a need for a UVH-specific PROM. To establish such a validated PROM, qualitative research using semi-structured and unstructured interviews is required to explore the full spectrum of chronic UVH symptoms.

Additionally, strong correlations were observed between the DHI physical and functional subscales (r = 0.74), as well as between the functional and emotional subscales (r = 0.72). Physical symptoms in UVH can adversely affect the ability to perform daily activities of patients. For instance, patients with chronic dizziness due to UVH may encounter difficulties in activities such as walking, climbing stairs, or driving. This can lead to significant limitations in daily functional domains such as work, school, or social activities. Additionally, these limitations may increase emotional stress and anxiety. It indicates that UVH is complex and multifaceted, affecting not only physical symptoms but also functional and emotional aspects of life.

### 4.10. Factors Related to QoL

Several factors were found to be related to QoL. Patients with PPPD reported decreased QoL among all outcome measures, which is consistent with the literature [92,93]. The decreased QoL in PPPD patients is likely due to the persistent and pervasive nature of dizziness symptoms triggered by common daily activities, such as standing, moving, or exposure to visual stimuli. These symptoms occur frequently, last for extended periods, and are challenging to avoid. Additionally, PPPD is characterized by its uncertain pathophysiology and limited treatment options, which further contribute to its negative impact on QoL. Furthermore, no association was found between the presence of migraine and QoL. This could be attributed to the assessment of patients during migraine-free periods [94,95].

### 4.11. Management of Chronic UVH

Although this study did not include data on previous treatments, addressing the management of chronic UVH is crucial. Management can involve different strategies, depending on the case: treatment of the underlying disorder (e.g., Menière’s Disease), treatment of secondary disorders (e.g., PPPD), and treatment of vestibular hypofunction. Regarding treatment of the underlying and secondary disorders, several new treatment options are currently investigated for, e.g., Menière’s Disease [96], Vestibular Migraine [97] and PPPD [98]. Regarding the treatment of chronic UVH, vestibular rehabilitation is the first choice [99]. However, not all patients show a significant response. For these patients, emerging strategies such as the use of a vestibular implant might be considered in the future [100,101].

### 4.12. Limitations

The inclusion criteria included vestibular symptoms and reduced responses on bithermal caloric tests. However, this selected patient group may not fully represent the entire UVH population, as the caloric test may overlook specific canal or otolith dysfunctions, resulting in a lower sensitivity [102]. Furthermore, reduced caloric responses may not always indicate a vestibular hypofunction, which can compromise specificity. Uncontrollable factors such as temporal bone anatomy can impede temperature conduction, which can possibly result in false-positive outcomes [102,103]. To overcome this factor as much as possible, laboratory-specific normative values were used. Finally, no specific analyses were performed on the presence of vestibular compensation status. After all, no world-wide consensus is yet present on the definition of a “compensated patient” (e.g., absence of spontaneous nystagmus, absence of asymmetry in rotatory chair testing, etc.). The sample size was also too small to add compensation status to the multiple linear regression analyses.

## 5. Conclusions

Chronic UVH is a heterogeneous disorder. It can result from many etiologies with different clinical presentations. UVH patients should be explicitly screened for secondary vestibular diagnoses such as BPPV and PPPD (and vice versa) because of their high prevalence. Furthermore, chronic UVH can significantly decrease QoL, especially in cases of co-existing PPPD. These findings highlight the need for a structured diagnostic approach and tailored interventions for chronic UVH patients. Interventions might vary from physical therapy to the treatment of co-existing vestibular and functional disorders, and novel strategies like the vestibular implant.

## Figures and Tables

**Table 1:** Characteristics of UVH patients.

Variables (*n* = 251)	Mean ± SD; (Range)
Age	58.96 ± 13.42; (18–84)
Gender	*n* (%)
Female	145 (58)
Male	106 (42)
Profession	
Employed	137 (55)
Unemployed	33 (13)
Retired	38 (15)
Missing	43 (17)
Duration of symptoms	
≥3–11 months	62 (25)
>12–23 months	50 (20)
≥24 months	139 (55)
The presence of attacks	
No attack	71 (28)
One attack	63 (25)
Previous attacks	44 (18)
Current attacks	73 (29)
Side of lesion	
Right	138 (55)
Left	113 (45)

Abbreviations: SD, standard deviation.

**Table 2:** MRI and CT findings in UVH patients.

Type			*n* (%)
MRI	Normal		127 (50.6)
*n* = 168	Abnormal	Vestibular schwannoma	17 (6.8)
		Vascular lesion	13 (5.2)
		Inner ear ossification/fibrosis	3 (1.2)
		Inner ear dysplasia	2 (0.8)
		Cerebral atrophy	2 (0.8)
		Other	4 (1.6)
CT	Normal		26 (10.4)
*n* = 37	Abnormal	SCDS	3 (1.2)
		Trauma/Skull base fracture	3 (1.2)
		Post-operative changes	2 (0.8)
		Other	3 (1.2)
Not tested			46 (18.3)
Total			251 (100)

Abbreviations: MRI, Magnetic Resonance Imaging; CT, computerized tomography; SCDS, superior canal dehissance syndrome.

**Table 3:** Questionnaire findings related to handicap level and quality of life in UVH patients.

	Handicap Level	Mild *n* (%)	Moderate *n* (%)	Severe *n* (%)	Mean ± SD
Quality of Life (*n* = 251)					
DHI Total		43 (17%)	118 (47%)	90 (36%)	51.63 ± 21.61
					Median (IQR)
HADS Depression		44 (18%)	40 (16%)	10 (4%)	6 (7.5)
HADS Anxiety		41 (16%)	42 (17%)	21 (8%)	6 (6.5)
EQ-5D-5L					VAS: 65 (30)

Abbreviations: DHI, dizziness handicap inventory; HADS, Hospital Anxiety and Depression Scale; EQ-5D-5L, EuroQoL–five dimensions–five levels; SD, standard deviation; IQR, interquartile range.

**Table 4:** Results from multiple linear regression analysis examining factors related to quality of life and psychological symptoms in UVH patients (n = 193).

Predictors	Baseline/Reference	Outcome Variable						
*n* = 193		DHI Total Score	DHI Physical Score	DHI Functional Score	DHI Emotional Score	HADS Depression Score	HADS Anxiety Score	EQ-5D-5L VAS Score
(Intercept)		47.04 ***	16.08 ***	16.53 ***	14.44 ***	6.68 ***	7.76 ***	66.29 ***
		(5.08)	(1.69)	(2.19)	(2.07)	(1.16)	(1.12)	(5.21)
PPPD	**Yes**	16.79 ***	5.40 ***	6.69 ***	4.70 **	2.07 *	2.41 **	−12.59 ***
	No	(3.51)	(1.17)	(1.51)	(1.43)	(0.80)	(0.78)	(3.59)
Migraine	**Yes**	4.68	0.56	2.24	1.88	1.02	0.30	−4.70
	No	(3.48)	(1.16)	(1.50)	(1.41)	(0.80)	(0.77)	(3.59)
Hearing	**Continuous variable**	−1.03	0.56 *	−0.27	−0.21	−0.11	−0.19	0.85
		(0.84)	(0.28)	(0.36)	(0.34)	(0.19)	(0.19)	(0.86)
The presence of attacks	**†**	F (3177) = 2.890, *p* = 0.136	**F (3177) = 3.136,** ***p* = 0.026**	F (3177) = 2.060, *p* = 0.107	F (3177) = 2.920, *p* = 0.135	F (3177) = 0.727, *p* = 0.536	**F (3177) = 2.185,** ***p* = 0.041**	F (3177) = 1.148, *p* = 0.331
	**One attack**	2.76	0.94	2.04	−0.22	−0.98	−1.66	−4.31
	No attack	(4.22)	(1.41)	(1.81)	(1.72)	(0.97)	(0.93)	(4.31)
	**Previous attacks**	−7.05	−3.40 *	−1.29	−2.36	−1.38	−1.93 *	3.62
	No attack	(4.30)	(1.44)	(1.85)	(1.75)	(0.99)	(0.95)	(4.42)
	**Current attacks**	4.23	−0.62	2.51	2.34	−0.90	−0.34	−2.08
	No attack	(3.85)	(1.28)	(1.66)	(1.57)	(0.88)	(0.85)	(3.97)
Duration of symptoms	**†**	F (2177) = 1.741, *p* = 0.178	F (2177) = 1.334, *p* = 0.024	F (2177) = 1.580, *p* = 0.208	F (2177) = 2.325, *p* = 0.100	F (2177) = 0.256, *p* = 0.774	F (2177) = 1.181, *p* = 0.329	F (2177) = 0.095, *p* = 0.908
	**12–24 months**	−2.25	0.79	−0.01	−3.03	−0.62	−0.45	0.17
	3–12 months	(4.25)	(1.42)	(1.83)	(1.73)	(0.98)	(0.94)	(4.34)
	**≥24 months**	4.34	1.80	2.23	0.31	−0.46	0.76	−1.23
	3–12 months	(3.36)	(1.12)	(1.44)	(1.37)	(0.77)	(0.74)	(3.43)
Vestibular test result	**†**	**F (2177) = 3.780,** ***p* = 0.024**	**F (2177) = 5.853,** ***p* = 0.003**	**F (2177) = 4.045,** ***p* = 0.019**	F (2177) = 0.490, *p* = 0.612	F (2177) = 2.224, *p* = 0.111	F (2177) = 1.059, *p* = 0.348	F (2177) = 1.449, *p* = 0.237
	**Caloric ≥ 6°/s and <25°/s**	−1.23	−1.07	−0.17	0.01	0.27	−0.28	0.78
	Caloric ≥25°/s	(3.60)	(1.20)	(1.55)	(1.46)	(0.83)	(0.80)	(3.72)
	**Caloric < 6°/s** Caloric ≥ 25°/s	10.85 *	3.90 *	5.18 *	1.76	2.30	1.12	−6.84
		(5.29)	(1.77)	(2.28)	(2.15)	(1.21)	(1.17)	(5.44)
R^2^		0.23	0.25	0.22	0.14	0.09	0.10	0.15
Adj. R^2^		0.19	0.21	0.17	0.09	0.04	0.04	0.10

Legend: Multiple linear regression analyses were conducted, incorporating data from 193 participants. The analyses examined the effects of six different predictors on distinct models of quality of life and symptoms. Each predictor’s baseline/reference value is indicated in regular font, while the values of interest for comparison are highlighted in bold. For “Hearing”, an increase in hearing scores corresponds to an increase in the degree of hearing loss. The † symbol denotes an omnibus test. Omnibus tests were conducted for factors with three or more categorical variables to assess differences between the groups. †: Omnibus test. *** *p* < 0.001; ** *p* < 0.01; * *p* < 0.05.

## Data Availability

The original contributions presented in the study are included in the article/Supplementary Material; further inquiries can be directed to the corresponding author.

## Supplementary Materials

The following supporting information can be downloaded at: https://www.mdpi.com/article/10.3390/jcm13185381/s1, Figure S1: Distribution of co-morbidities in the UVH population; Figure S2: Distribution of DISCOHAT symptoms in the UVH population; Figure S3: Scatterplot illustrating the correlation between caloric asymmetry and vHIT asymmetry in the UVH population; Figure S4: Scatterplot illustrating the correlation between caloric asymmetry and questionnaires related to QoL in the UVH population; Figure S5: Scatterplot illustrating the correlation between vHIT asymmetry and questionnaires related to QoL in the UVH population; Figure S6: Scatterplot illustrating the correlation between questionnaires related to QoL in the UVH population; Table S1: Etiologies and primary diagnoses of UVH; Table S2: Results of pure tone audiometry.
