# Supplementary material for: Chronic Unilateral Vestibular Hypofunction: Insights into Etiologies, Clinical Subtypes, Diagnostics and Quality of Life
Source: J Clin Med. 2024 Sep 11;13(18):5381. doi: 10.3390/jcm13185381 (PMC11432443; doi:10.3390/jcm13185381)

**Table S1.** Etiologies and primary diagnoses of UVH

| **Etiologies** | **Diagnoses** | N | % |
|---|---|---|---|
| Menière's Disease | Menière's Disease | 79 | 31 |
| Infection/Inflammation | Acute unilateral vestibulopathy/ Vestibular neuritis | 35 | 14 |
| | Labyrinthitis | 9 | 4 |
| Neoplasm | Vestibular schwannoma | 17 | 7 |
| | Intrameatal lipoma | 1 | 0.5 |
| Vestibular Migraine | Vestibular Migraine | 17 | 7 |
| Overlapping MD/VM | Overlapping MD/VM | 11 | 4 |
| Trauma | Labyrinthine concussion | 1 | 0.5 |
| | Skull base fracture | 8 | 3 |
| Iatrogenic | Stapedotomy | 5 | 2 |
| | Cholesteatoma | 1 | 0.5 |
| | Myringo/tympanoplasty | 1 | 0.5 |
| Autoimmune disorder | Autoimmune inner ear disease | 6 | 2 |
| Benign recurrent vertigo | Benign recurrent vertigo | 5 | 2 |
| Congenital | Congenital inner ear malformations | 4 | 2 |
| Vascular | Inner ear ischemia | 3 | 1 |
| Otosclerosis | Otosclerosis | 2 | 1 |
| Idiopathic/Unknown | Idiopathic/Unknown | 46 | 18 |
| Total | | 251 | 100 |

**Table S2.** Results of pure tone audiometry

| Hearing status | FI (dB) | Asymmetric hearing | | Symmetric hearing | |
|---|---|---|---|---|---|
| | | **Best ear** | **Worst ear** | **Best ear** | **Worst ear** |
| Normal hearing | -10 to 15 | 47 | 0 | 47 | 40 |
| Slight hearing loss | 16 to 25 | 33 | 5 | 24 | 23 |
| Mild hearing loss | 26 to 40 | 27 | 19 | 21 | 24 |
| Moderate hearing loss | 41 to 55 | 12 | 29 | 7 | 12 |
| Moderately severe hearing loss | 56 to 70 | 4 | 36 | 8 | 7 |
| Severe hearing loss | 71 to 90 | 1 | 24 | 2 | 1 |
| Profound hearing loss | 91+ | 0 | 11 | 5 | 7 |
| Not tested | 13 | | | | |
| Total | 251 | 124 | 124 | 114 | 114 |
| **Abbreviations:** FI, Fletcher Index | | | | | |

**Figure S1.** Distribution of co-morbidities in the UVH population

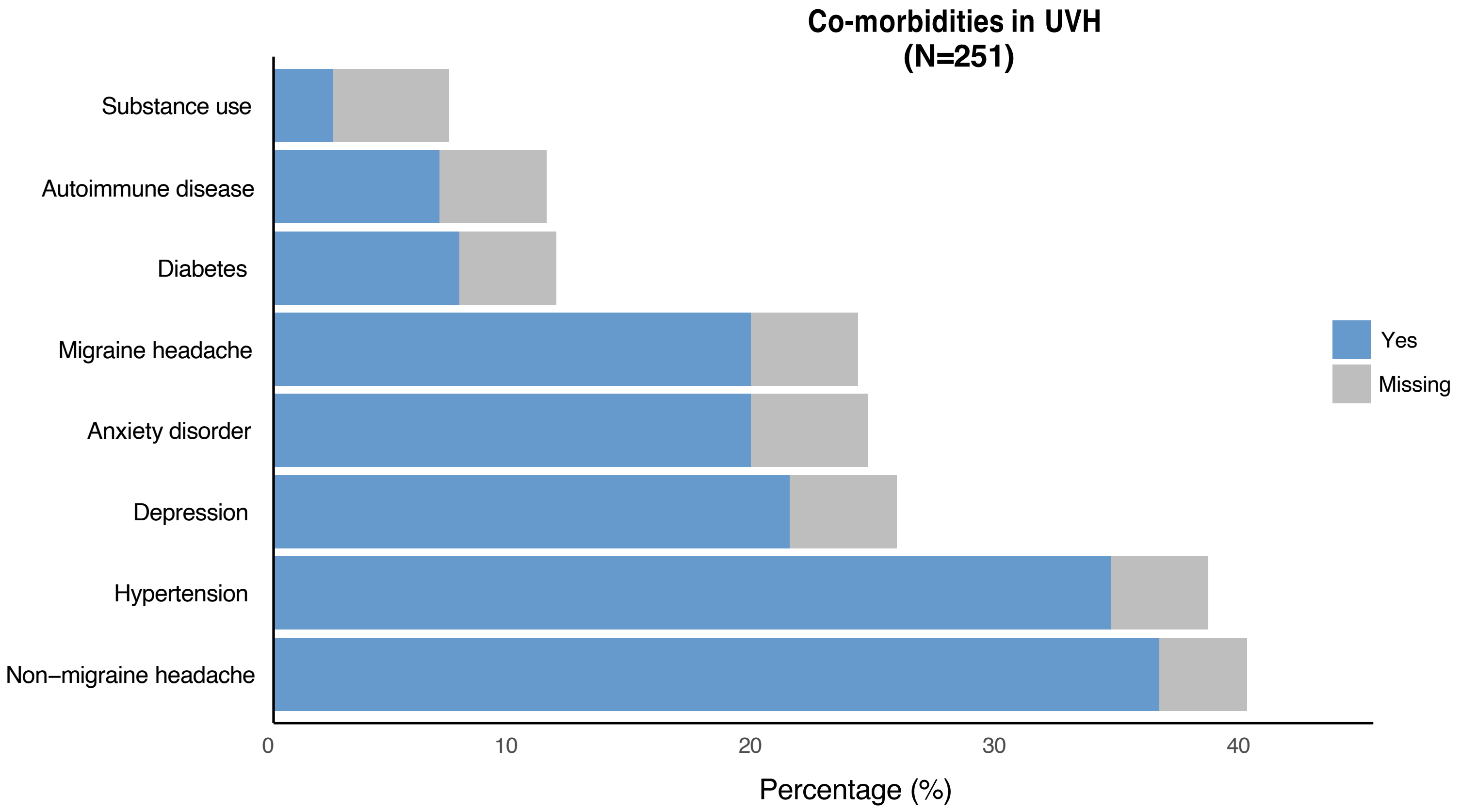

**Figure S2.** Distribution of DISCOHAT symptoms in the UVH population

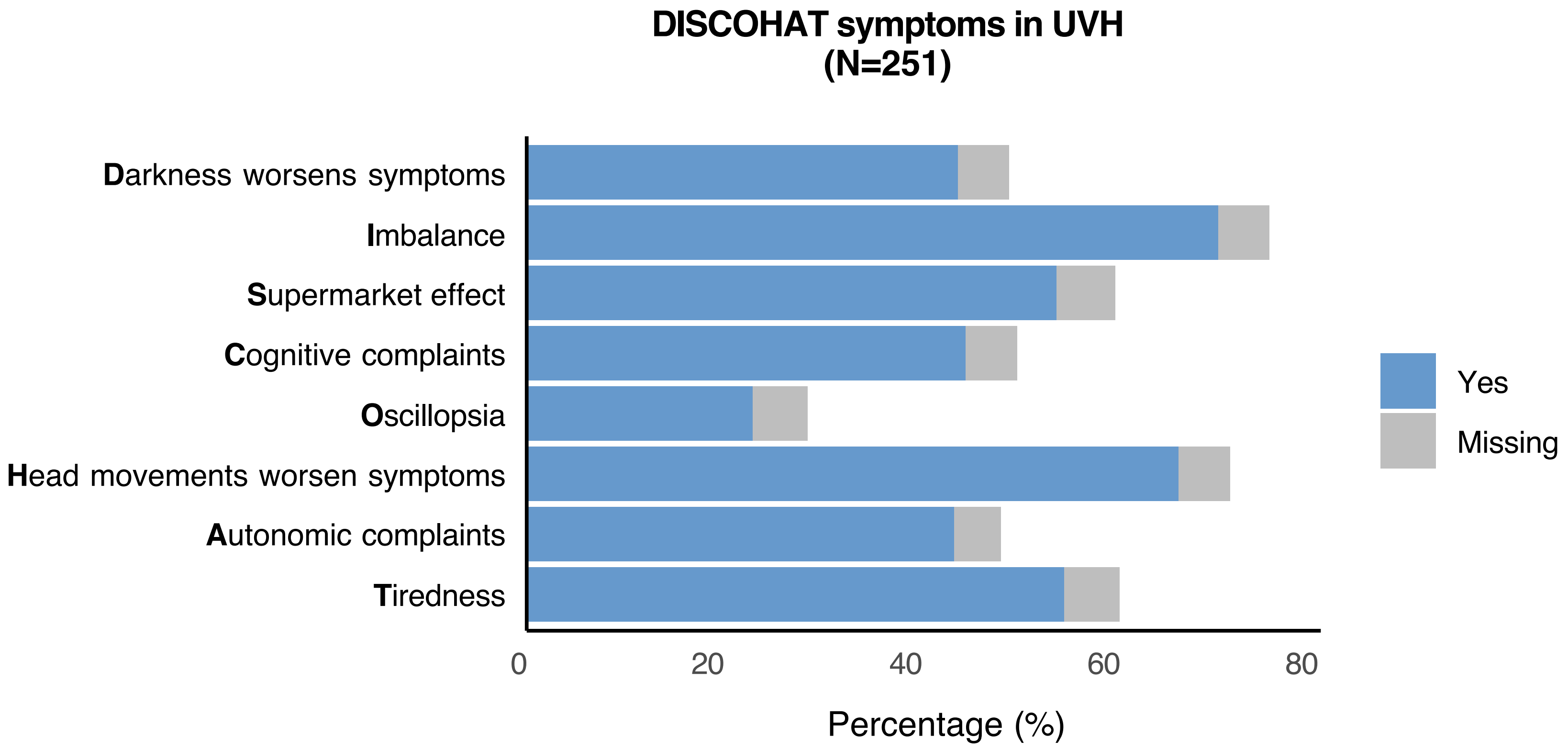

**Figure S3.** Scatterplot illustrating the correlation between caloric asymmetry and vHIT asymmetry in the UVH population

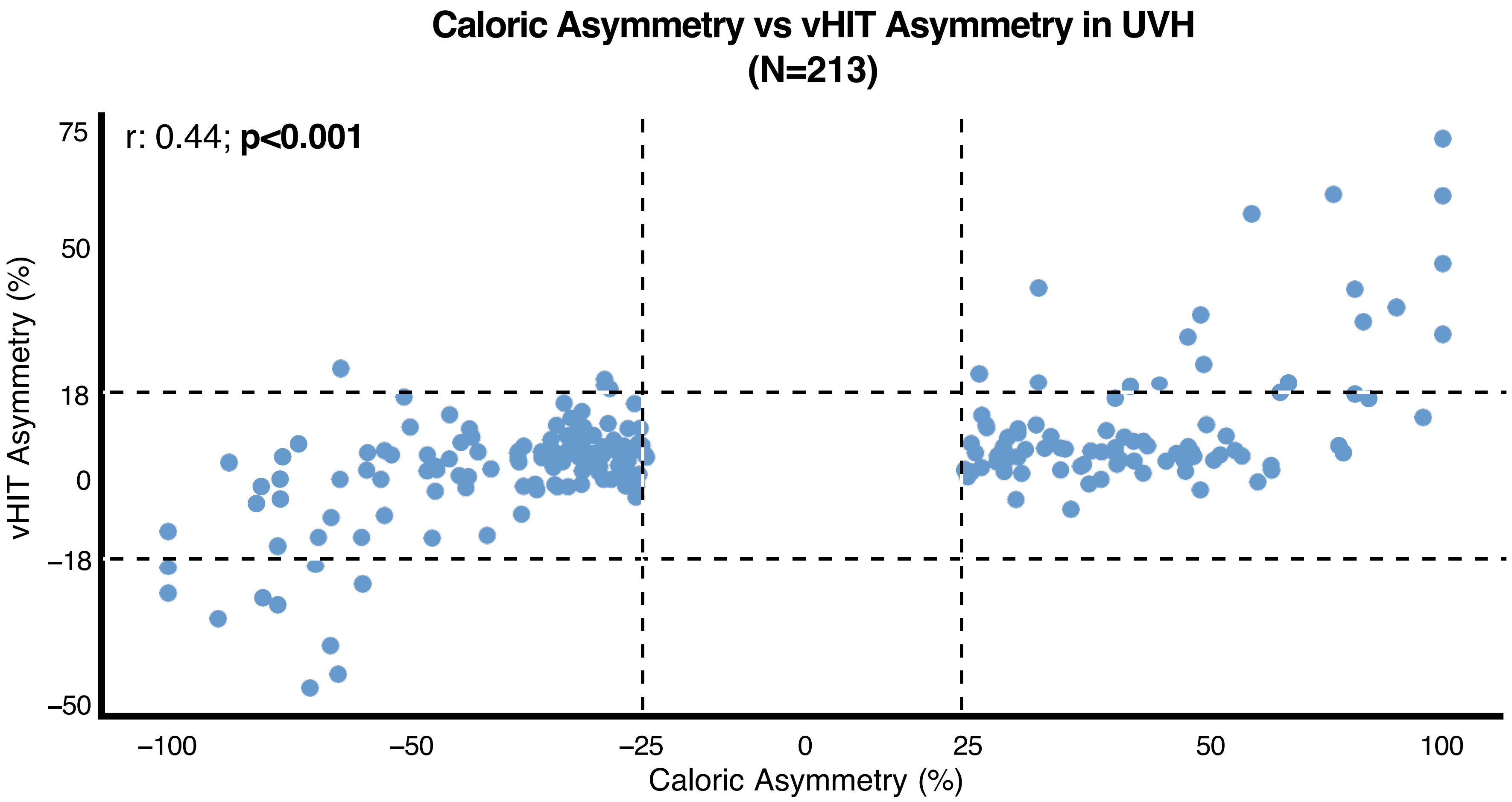

**Figure S4.** Scatterplot illustrating the correlation between caloric asymmetry and questionnaires related to QoL in the UVH population

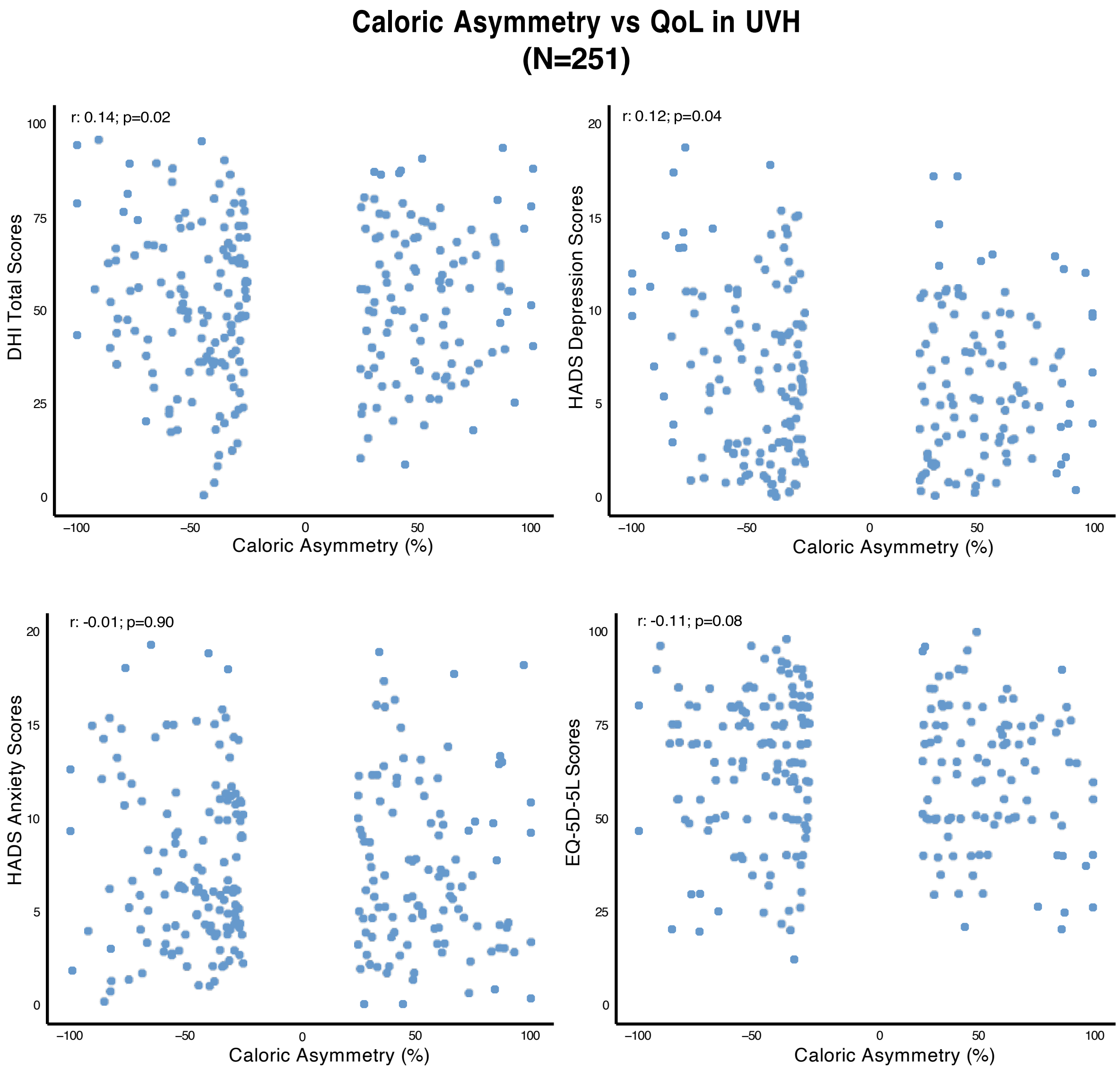

**Figure S5.** Scatterplot illustrating the correlation between vHIT asymmetry and questionnaires related to QoL in the UVH population

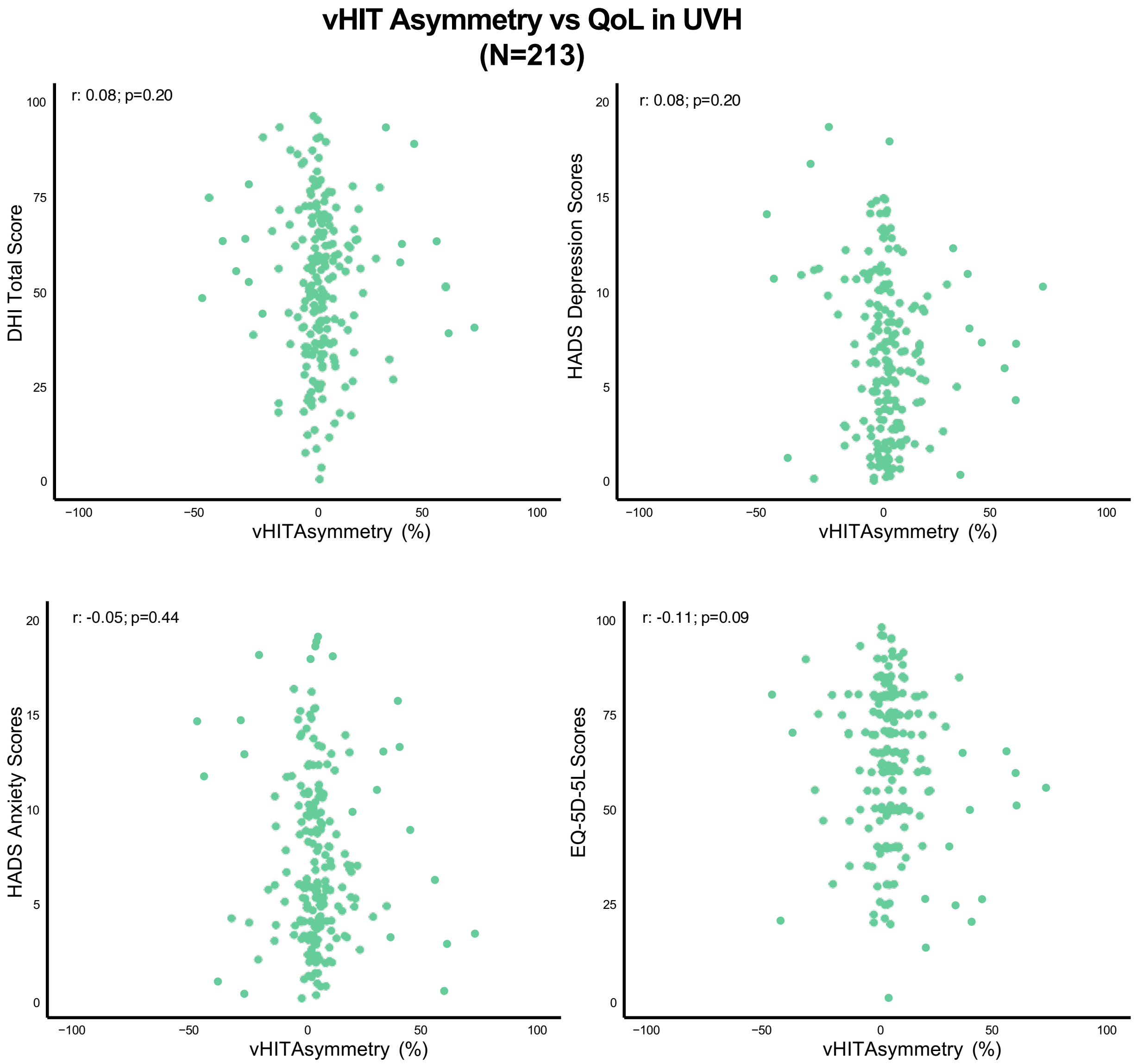

**Figure S6.** Scatterplot illustrating the correlation between questionnaires related to QoL in the UVH population

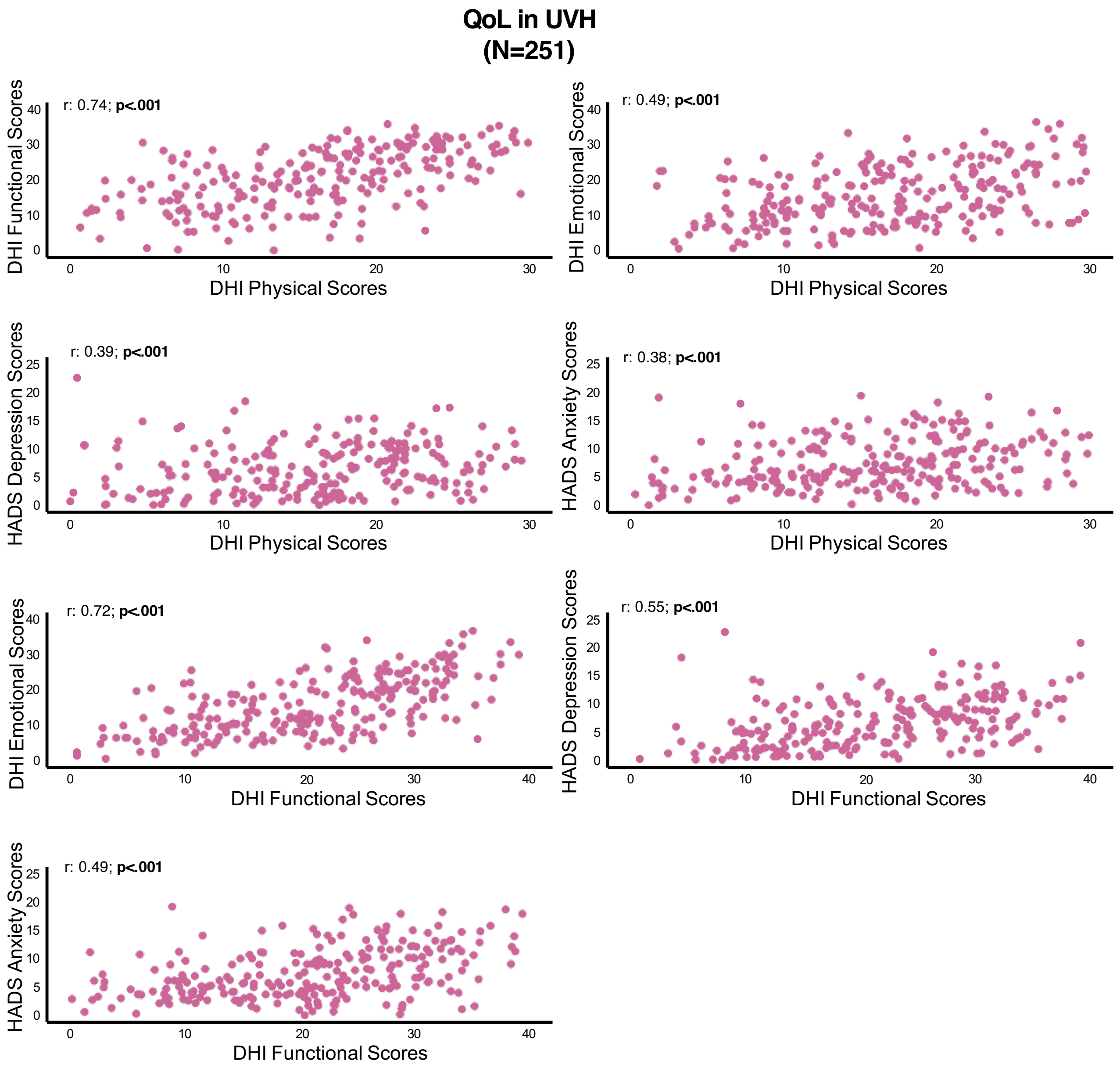

Supplement: Supplementary file 1 [file jcm-13-05381-s001.zip › jcm-3175785-supplementary.pdf]
